# A new reservoir-based CPAP with low oxygen consumption: the Bag-CPAP

**DOI:** 10.1186/s13054-023-04542-2

**Published:** 2023-07-04

**Authors:** Eloïse de Beaufort, Guillaume Carteaux, François Morin, Arnaud Lesimple, Anne-Fleur Haudebourg, Emeline Fresnel, Damien Duval, Alexandre Broc, Alain Mercat, Laurent Brochard, Dominique Savary, François Beloncle, Armand Mekontso Dessap, Jean-Christophe Richard

**Affiliations:** 1 Université Paris Est-Créteil, Faculté de Santé, Groupe de Recherche Clinique CARMAS, 94010 Créteil, France; 2 Med2Lab Laboratory, ALMS, Antony, France; 3 Assistance Publique-Hôpitaux de Paris, CHU Henri Mondor, Service de Médecine Intensive Réanimation, 1 rue Gustave Eiffel, 94010 Créteil Cedex, France; 4 INSERM U955, Institut Mondor de Recherche Biomédicale, 94010 Créteil, France; 5 Centre Hospitalier Universitaire d’Angers, Département de Médecine d’Urgence, Université d’Angers, Faculté de Santé, Vent’Lab, Angers, France; 6 CNRS, INSERM 1083, MITOVASC, Université d’Angers, Angers, France; 7 Kernel Biomedical, Bois-Guillaume, France; 8 Centre Hospitalier Universitaire d’Angers, Département de Médecine Intensive-Réanimation et Médecine Hyperbare, Vent’Lab, Université d’Angers, Faculté de Santé, Angers, France; 9 Keenan Research Centre, Li Ka Shing Knowledge Institute, St. Michael’s Hospital, Toronto, Canada; 10 Interdepartmental Division of Critical Care Medicine, University of Toronto, Toronto, Canada; 11 INSERM, UMR 1066, Créteil, France

**Keywords:** Oxygen consumption, Acute hypoxemic respiratory failure, Continuous positive airway pressure, Frugal innovation

## Abstract

**Background:**

Several noninvasive ventilatory supports rely in their design on high oxygen consumption which may precipitate oxygen shortage, as experienced during the COVID-19 pandemic. In this bench-to-bedside study, we assessed the performance of a new continuous positive airway pressure (CPAP) device integrating a large reservoir (“Bag-CPAP”) designed to minimize oxygen consumption, and compared it with other CPAP devices.

**Methods:**

First, a bench study compared the performances of Bag-CPAP and four CPAP devices with an intensive care unit ventilator. Two FiO_2_ targets (40–60% and 80–100%) at a predefined positive end expiratory pressure (PEEP) level between 5 and 10 cm H_2_O were tested and fraction of inspired oxygen (FiO_2_) and oxygen consumption were measured. Device-imposed work of breathing (WOB) was also evaluated. Second, an observational clinical study evaluated the new CPAP in 20 adult patients with acute respiratory failure in two hospitals in France. Actual FiO_2_, PEEP, peripheral oxygen saturation, respiratory rate, and dyspnea score were assessed.

**Results:**

All six systems tested in the bench study reached the minimal FiO_2_ target of 40% and four reached at least 80% FiO_2_ while maintaining PEEP in the predefined range. Device-delivered FiO_2_/consumed oxygen ratio was the highest with the new reservoir-based CPAP irrespective of FiO_2_ target. WOB induced by the device was higher with Bag-CPAP. In the clinical study, Bag-CPAP was well tolerated and could reach high (> 90%) and moderate (> 50%) FiO_2_ with an oxygen flow rate of 15 [15–16] and 8 [7–9] L/min, respectively. Dyspnea score improved significantly after introduction of Bag-CPAP, and SpO_2_ increased.

**Conclusions:**

In vitro, Bag-CPAP exhibited the highest oxygen saving properties albeit had increased WOB. It was well accepted clinically and reduced dyspnea. Bag-CPAP may be useful to treat patients with acute respiratory failure in the field, especially when facing constraints in oxygen delivery.

**Supplementary Information:**

The online version contains supplementary material available at 10.1186/s13054-023-04542-2.

## Background

The recent COVID-19 pandemic subjected the healthcare systems, around the globe, to unprecedented challenges brought by the massive influx of critically ill patients with acute hypoxemic respiratory failure (AHRF) that exceeded hospital capacity [1]. Not all patients had access to ventilatory support due to the combined shortage of artificial ventilators and of professionals trained on the use of ventilatory support and oxygen supply. For such, many healthcare professionals tried to avoid intubation and spare more intensive care unit (ICU) beds by using continuous positive airway pressure (CPAP) as a backup treatment solution to provide noninvasive oxygenation and respiratory support outside ICU [2].

Most of the current noninvasive ventilatory support devices consume big amounts of oxygen to achieve optimal inspired oxygen fraction (FiO_2_), and that may disrupt healthcare organization and add pressure on hospital oxygen delivery capabilities [3]. The COVID-19 crisis highlighted the need for oxygen-sparing respiratory support devices. The constraint of oxygen delivery is also structural in the vast majority of low-middle income countries where most of the critically-ill patients on earth reside [4].

CPAP is a simple respiratory support, easy to train healthcare personnel on, and easy to deliver without an artificial ventilator using inexpensive devices that require only an oxygen source [5]. Interestingly, Perkins et al*.* reported that CPAP may reduce the need for tracheal intubation and the risk of mortality, compared with conventional oxygen therapy, in patients with COVID-19 induced AHRF [6].

We hypothesized that the currently, commercially available CPAP devices did not have equal performances in terms of optimal respiratory support to deliver and oxygen requirements to fulfil. Within the framework of frugal innovation [3, 4], we designed a reservoir-based CPAP, the so-called “Bag-CPAP”, to match positive pressure with oxygen needs in conditions where oxygen flow is limited (5–15 L/min), and to maintain acceptable performances when oxygen is not limited. The purpose of the present bench to bedside study was to address the following questions: First, can Bag-CPAP efficiently deliver predefined FiO_2_ targets with minimal oxygen requirements while ensuring adequate PEEP, as compared with available CPAP devices? Second, is Bag-CPAP efficient in treating patients with AHRF in terms of FiO_2_, airway pressure (Paw), and clinical tolerance?

## Materials and methods

### The Bag-CPAP device

**Fig. 1:**
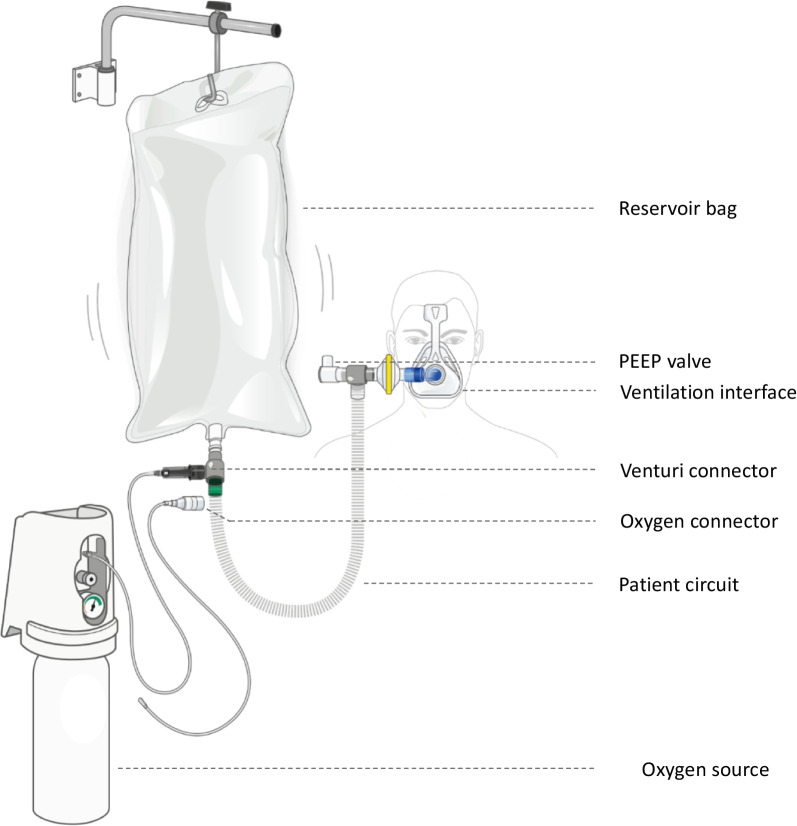
Description of Bag-CPAP. The figure presents Bag-CPAP. The system is composed of the following elements: (i) a connector with a Venturi system (providing the gas at 50% FiO_2_ from an oxygen source of 5 L/min, even from an extractor), or a conventional oxygen connector without Venturi (providing the gas at > 90% FiO_2_ from an oxygen source of 15 L/min); (ii) a 30L reservoir to cover patients respiratory demand; (iii) two 3D printed pieces (safety pressure valve and non-rebreathing valve) to connect the patient circuit to the reservoir and to the patient mask; (iv) a positive expiratory pressure valve to deliver PEEP between 5 and 10 cm H_2_O

The design of the Bag-CPAP device (Air Liquide Medical Systems, Antony, France) is provided in Fig. 1. Its aim is to deliver PEEP at 5, 7.5, or 10 cm H_2_O by attaching appropriate interchangeable expiratory valves. The system operates with a 30 L reservoir (Bag) in which oxygen is accumulated to control its consumption and to guarantee attaining the targeted FiO_2_ level irrespective of the patient’s respiratory demand. Two levels of FiO_2_ can be delivered by selecting two interchangeable connectors: moderate FiO_2_ (50–60%), obtained with an oxygen flow rate of at least 5 L/min using a Venturi system; or high FiO_2_ (90–100%), obtained with an oxygen flow rate of at least 15 L/min using a conventional connector. If oxygen is not limited, its flow rate can be increased to 10 L/min with the Venturi system and up to 30 L/min with the conventional connector to minimize the device-induced WOB. A non-rebreathing valve is positioned close to the mask, allowing the patient to inhale moderate or high FiO_2_ during the inspiratory phase and to exhale through the PEEP valve during the expiratory phase, while oxygen accumulates in the bag. An anti-asphyxia valve is also integrated inside the PEEP valve to comply with high patient inspiratory demand.

### Bench assessment

#### Devices

The performances of Bag-CPAP and of four other noninvasive devices were compared with those of an ICU ventilator (Puritan Bennett 980™; Covidien, Dublin, Ireland). The four tested devices were: one homecare CPAP ventilator (AirSense™ 10 AutoSet™; Resmed, Saint Priest, France); two virtual valve CPAP devices (Boussignac CPAP; Vygon, Ecouen, France, and O-two CPAP; O-two, Brampton, Canada), and one CPAP mask with a Venturi system (StarMed™ Ventumask CPAP; Intersurgical, Wokingham, United Kingdom). These devices were chosen to cover a large range of CPAP mechanisms available for patients with AHRF. Characteristics of each device are summarized in Additional file 1: Table S1. The PB 980 ventilator was used as the gold standard in bench test evaluation.

#### FiO_2_ evaluation

##### Set-up

**Fig. 2:**
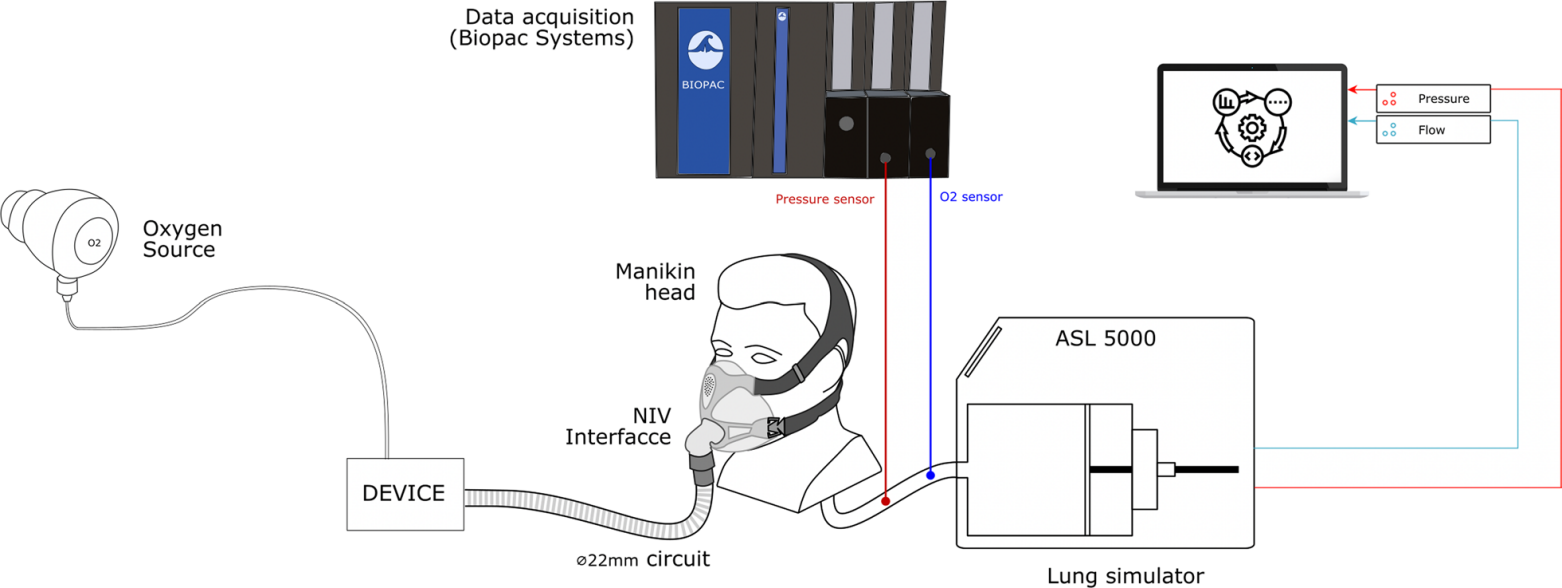
Description of the experimental bench model. The figure presents the bench set-up used to evaluate the different CPAP devices in terms of FiO_2_ and oxygen consumption. All devices were connected to a manikin head with realistic upper airways structures. The manikin’s trachea was connected to the ASL 5000 test lung to simulate spontaneous breathing. A pressure transducer and an oxygen sensor were used to measure the airway pressure and the inspired fraction of oxygen, respectively, and were placed in the trachea of the manikin. All signals were recorded using an analog/numeric data-acquisition system (Biopac systems)

The bench set-up is illustrated in Fig. 2. All devices were tested on a manikin head with realistic upper airways structure (Kernel Biomedical, Bois-Guillaume, France) [7]. The head comprised a removable mandible, flexible airway parts (pharynx and trachea), and a silicone skin, all fixed on a base. The dead space is 152 mL and the airways resistance is 2.4 cm H_2_O/L/s. The head can work either with the mouth open or closed.

The manikin’s trachea was connected to ASL 5000^®^ (IngMar Medical, Pittsburg, PA, USA) test lung to simulate spontaneous breathing. A pressure transducer (SD160 series: Biopac systems, Goleta, CA, USA) and a paramagnetic-based oxygen measurement module (O2100C: Biopac systems, Goleta, CA, USA) were placed in the trachea to measure Paw and FiO_2_, respectively. Generated signals were recorded at 2000 Hz using an analog/numeric data-acquisition system (MP150, Biopac systems, Goleta, CA, USA) and analyzed with Acqknowledge software version 5.0 (Biopac systems, Goleta, CA, USA). Flow and volume readings were collected from the ASL 5000 software at 512 Hz.

##### Simulated patient profiles

Two patient profiles were simulated to mimic combinations of oxygenation needs and respiratory mechanics:

- Moderate respiratory demand: respiratory system resistance (*R*_RS_) and compliance (*C*_RS_) were set at 6 cm H_2_O/L/s and 60 mL/cm H_2_O, respectively. The respiratory rate was set at 20 cycle per minute (cpm). The muscle pressure (Pmus) was set on ASL to reach a tidal volume of around 550 ml for each tested device.
- High respiratory demand:* R*_RS_ was set at 6 cm H_2_O/L/s,* C*_RS_ at 30 mL/cm H_2_O, and respiratory rate at 30 cpm. The muscle pressure (Pmus) was set on ASL to reach a tidal volume of around 650 ml for each tested device.

For both respiratory demands, a Pmus periodic variation of ± 15% was applied on the active lung model to reproduce spontaneous breathing variability.

##### Protocol

Each device was assessed based on two different FiO_2_ target ranges adapted to the patient profile severity: FiO_2_ range of 40–60% for the moderate respiratory demand and FiO_2_ range of 80 to 100% for the high respiratory demand. Oxygen flow rates were adjusted on each device, according to manufacturer’s recommendations, to reach those targets with the aim of delivering a PEEP level between 5 and 10 cm H_2_O (which may depend on the oxygen supply for open valve systems). This PEEP range was chosen as it represents the most widely used range of CPAP in clinical practice [8, 9].

##### FiO_2_ and volume averaged FiO_2_

For all devices, mean FiO_2_ was analyzed for the two respiratory demands. The volume averaged FiO_2_ was computed to assess the effective FiO_2_ delivered to the lungs, taking into account variations of inspiratory flow rate and FiO_2_ throughout the inspiratory time (see Additional file 1: Fig. S1) [10, 11]. After testing each device, the ASL 5000 test lung was washed with fresh air until the FiO_2_ returned to 21% (± 0.1).

To assess the efficiency of oxygen delivery for each device, the ratio of averaged FiO_2_ to oxygen flow rate was calculated for all noninvasive devices (the oxygen flow rate was not available for the ICU ventilator). A high ratio indicated a CPAP capable of achieving high FiO_2_ with low oxygen consumption.

#### Inspiratory resistive load and work of breathing (WOB) added by the device

The maximum change between inspiratory and expiratory airway pressure was measured, and expressed as peak-to-peak airway pressure (P-P) [12]. For each CPAP device, the relative change in WOB was calculated and expressed as a percentage of WOB measured with the ICU ventilator. Additionally, dynamic Pmus–volume curves were used to assess the increase in the simulated patient’s WOB required to maintain the targeted tidal volume.

Each device was connected to the ASL 5000 test lung to quantify the device-related inspiratory resistive load generated in two simulated efforts. Pmus, Paw, and volume signals were recorded from the ASL 5000 software at 512 Hz.

Each device was tested for the two respiratory demands by adapting Pmus on ASL (with* R*_RS_ = 6 cm H_2_O/L/s,* C*_RS_ = 60 mL/cm H_2_O, respiratory rate = 20 cpm) in order to reach a tidal volume of around 300 mL or around 500 mL, accordingly. PEEP level was adjusted at 7 (± 1) cm H_2_O and oxygen flow rate was set to obtain 40–60% FiO_2_ for each device. Bag-CPAP was evaluated with different oxygen flow rates: 5 L/min and 10 L/min with the Venturi connector, and 15 L/min and 20 L/min with the conventional connector. In addition, the oxygen flow needed to obtain a comparable WOB with Bag-CPAP and Boussignac CPAP was assessed.

### Clinical observational study

A special temporary authorization to use Bag-CPAP in usual care on 20 patients was delivered by the French National Agency for the Safety of Medicines and Health Products (*Agence Nationale de Sécurité du Médicament et des produits de santé*, ANSM). The clinical data of these patients collected from routine care were used in our study, which was approved by the ethics committee (CE SRLF 21-102) of the French Intensive Care Society (*Société de Réanimation de Langue Française*). Patients were eligible if they had developed AHRF requiring more than 5 L/min of oxygen. Oxygenation and ventilation modalities preceding the initiation of Bag-CPAP are summarized in Table 1: 17 patients had a face mask reservoir bag, two patients received oxygen through a HFOT device and one underwent NIV with bilevel pressure [13]. The consent process was as follows: patients received oral and written information and were included in case of non-opposition as per the French law regarding observational studies. The study was conducted at Henri-Mondor (ICU) and Angers (emergency department and ICU) university hospitals (France).

**Table 1:** Oxygenation and ventilation modalities preceding the initiation of Bag-CPAP

Device	Oxygen flow	Previous FiO_2_ (%)	SpO_2_ (%)	Etiology
High concentration mask	15 L/min	90–100*	100	Acute cardiogenic pulmonary edema
High concentration mask	15 L/min	90–100*	91	Acute cardiogenic pulmonary edema
High concentration mask	6 L/min	60*	N/A	SARS-CoV-2
High concentration mask	10 L/min	80–90*	95	Acute decompensation of interstitial lung disease
High concentration mask	15 L/min	90–100*	86	SARS-CoV-2
High concentration mask	9 L/min	80–90*	98	Acute decompensation of interstitial lung disease
High concentration mask	15 L/min	90–100*	84	SARS-CoV-2
High concentration mask	15 L/min	90–100*	95	SARS-CoV-2
High concentration mask	6 L/min	60*	96	SARS-CoV-2
High concentration mask	15 L/min	90–100*	92	SARS-CoV-2
High concentration mask	15 L/min	90–100*	91	SARS-CoV-2
High concentration mask	15 L/min	90–100*	94	SARS-CoV-2
NIV	N/A	60	99	Acute cardiogenic pulmonary edema
High concentration mask	9 L/min	80–90*	97	SARS-CoV-2
High concentration mask	10 L/min	80–90*	90	SARS-CoV-2
High concentration mask	10 L/min	80–90*	93	SARS-CoV-2
HFOT	N/A	93	94	SARS-CoV-2
HFOT	N/A	100	88	SARS-CoV-2
High concentration mask	6 L/min	60*	98	Acute cardiogenic pulmonary edema
High concentration mask	15 L/min	90–100*	86	SARS-CoV-2

*FiO_2_ estimated according to O’Driscoll BR, Howard LS, Earis J, Mak V. BTS guideline for oxygen use in adults in healthcare and emergency settings. Thorax. 2017;72:ii1–90

HFOT = high flow oxygen therapy

#### Bag-CPAP implementation

For the purpose of this first clinical use, Bag-CPAP was implemented according to the following pragmatic recommendations:

- The 7.5 cm H_2_O single use PEEP valve was used by default but could be changed, based on the clinical response, to a 5 cm H_2_O or a 10 cm H_2_O PEEP valve.
- The “moderate FiO_2_” connector was used if the patient needed ≤ 10 L/min oxygen therapy to maintain SpO_2_ at ≥ 95% before Bag-CPAP initiation.
- The “high FiO_2_” connector was used if the patient needed > 10 L/min oxygen therapy to maintain SpO_2_ at ≥ 95% before Bag-CPAP initiation.

It was possible to adjust FiO_2_ according to patient’s monitored SpO_2_ whilst being on Bag-CPAP treatment. As per clinical routine, the first Bag-CPAP session was continued for at least 4 h. In case of poor tolerance, Bag-CPAP was interrupted and replaced by another support, at the discretion of the attending physician. Standard respiratory and hemodynamic data, including dyspnea assessment using the modified Borg dyspnea scale, were collected at baseline, within the ten minutes of Bag-CPAP initiation, and at 30, 60, 120, 180, and 240 min after Bag-CPAP initiation, along with both FiO_2_ and Paw into the mask, recorded with an oxygen analyzer MX300 (Teledyne Analytical Instruments) and a standard manometer, respectively.

#### Endpoints

The clinical study aimed at testing the efficacy (notably in terms of oxygenation) and safety of Bag-CPAP device in clinical practice. The efficacy endpoints included actual FiO_2_ delivered to patient, oxygen flow rate, Paw (CPAP level), SpO_2_, respiratory rate, and modified Borg dyspnea scale.

### Statistics

Quantitative data were expressed as mean (standard deviation) or median (interquartile range) depending on whether the distribution was normal or not. Normality of the distribution was assessed using Shapiro Wilk test. In the bench study, where variables were normally distributed, devices were compared using one-way ANOVA with Bonferroni correction. In the clinical study, where variables were non-normally distributed, paired quantitative data were compared by Wilcoxon test. Qualitative data were compared by Chi-2 test or Fisher's exact test. Tests were two-tailed and a value of *P* < 0.05 was considered statistically significant. Statistical analysis was performed using SPSS Base 29 (SPSS Inc, Chicago, IL).

## Results

### Bench study

#### FiO_2_

**Fig. 3:**
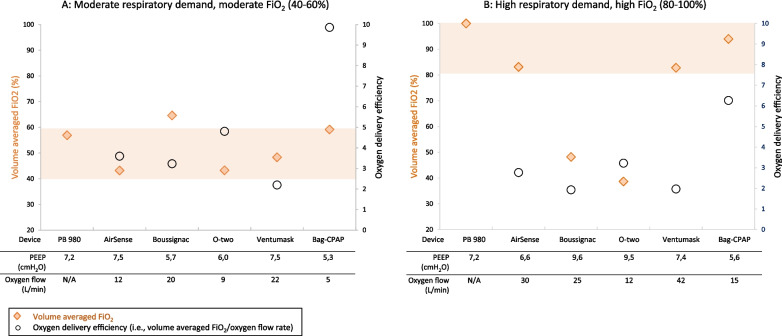
Oxygen delivery efficiency of the different devices to test. Oxygen delivery efficiency was assessed for noninvasive devices at two FiO_2_ target ranges corresponding to two clinical scenarios: 40–60% while the test lung simulated a moderate respiratory demand (panel **A**) and 80–100% while the test lung simulated a high respiratory demand (panel **B**), see methods section for more details. Oxygen flow rates were thus adjusted on each device according to manufacturer’s recommendations to reach those two FiO_2_ target ranges with the aim of delivering a PEEP level between 5 and 10 cm H_2_O. The areas shaded in orange represent the two FiO_2_ target ranges. The orange diamonds represent the value of the volume averaged FiO_2_ (i.e. the FiO_2_ actually delivered into the lungs in inspiration). The circles represent the oxygen delivery efficiency calculated as volume averaged FiO_2_/oxygen flow rate ratio; the greater this ratio, the more efficient the device is in terms of oxygen delivery and oxygen saving properties

Under the simulated moderate respiratory demand, the target ranges for PEEP (5–10 cm H_2_O) and FiO_2_ (40–60%) were achieved with all tested devices except the Boussignac [for which FiO_2_ was > 60%, as 20L/min oxygen flow rate was needed to achieve the target PEEP] (Fig. 3—panel A). Under the simulated high respiratory demand, O-two and Boussignac failed to reach the high FiO_2_ target range (80–100%) (Fig. 3—panel B). Bag-CPAP had the highest oxygen delivery efficiency of all noninvasive devices in both simulated clinical scenarios, i.e., required the lowest oxygen flow rate to reach the FiO_2_ target (Table 2). FiO_2_ measured at the trachea of the manikin provided slightly different results compared with volume averaged FiO_2_ computation (Fig. 2 and Additional file 1: Fig. S2).

**Table 2:** Oxygen consumption and actual Fio_2_ delivered by each device in two case scenarios

	Moderate respiratory demand—Moderate FiO_2_ (40–60%)		High respiratory demand—High FiO_2_ (80–100%)	
	Oxygen flow (L/min)	Mean FiO_2_ (%)	Oxygen flow (L/min)	Mean FiO_2_ (%)
Puritan Benett^a^	N/A	56 ± 0.5	N/A	100 ± 0.02
AirSense CPAP	12	48 ± 0.4	30	84 ± 0.9
Boussignac CPAP	20	66 ± 1.8	25	50 ± 0.8
O-two CPAP	9	42 ± 0.7	12	38 ± 0.3
Ventumask CPAP^b^	22	47 ± 0.6	42	80 ± 1.9
Bag-CPAP	5	60 ± 0.5	15	96 ± 1.3

Moderate and high respiratory demands correspond to two simulated patient efforts, detailed in the methods section. Each device was assessed with two different FiO_2_ target ranges depending on the simulated demand: moderate FiO_2_ (40–60%) for the moderate respiratory demand, and high FiO_2_ (80–100%) for the high respiratory demand. Mean (standard deviation) FiO_2_ was calculated as the mean fraction of oxygen measured at the trachea of the manikin during the simulated respiratory cycle

^a^Oxygen flow rate was not available with the ICU ventilator, moderate FiO_2_ was set at 55% and high FiO_2_ at 100%

^b^Ventumask was used with two oxygen sources (tube A and tube B, according to manufacturer’s specifications)

#### Resistive load

Maximum change between inspiratory and expiratory airway pressure, expressed as peak-to-peak airway pressure (P-P), is reported in Table 3. All differences between devices were statistically significant. AirSense CPAP exhibited the lowest P-P (0.9 and 1.7 cm H_2_O for moderate and high demand, respectively), while Ventumask and Bag-CPAP with the Venturi connector at 5 L/min of oxygen, exhibited the highest P-P value for moderate demand (5.9 and 7.8 cm H_2_O, respectively) and for high demand (11.8 and 11.2 cm H_2_O, respectively).

**Table 3:** Devices inspiratory resistive load

	O_2_ flow (L/min)	PEEP (cm H_2_O)	Inspiratory demand with Vt target of 300 mL	Inspiratory demand with Vt target of 500 mL
			P-P (cm H_2_O)*	P-P (cm H_2_O)*
PB 980	N/A	6.0	4.1 ± 0.05	5.2 ± 0.02
AirSense CPAP	N/A	6.8	0.9 ± 0.02	1.7 ± 0.03
Boussignac CPAP	20	6.6	3.0 ± 0.05	4.9 ± 0.04
O-Two CPAP	9	7.1	4.0 ± 0.05	6.6 ± 0.06
Ventumask CPAP	22	6.6	5.9 ± 0.05	11.8 ± 0.02
Bag-CPAP Venturi connector	5	5.9	7.8 ± 0.03	11.2 ± 0.02
	10	7.5	1.4 ± 0.02	2.9 ± 0.03
Bag-CPAP conventional connector	15	6.2	5.6 ± 0.04	9.2 ± 0.02
	20	5.7	4.0 ± 0.06	8.7 ± 0.05

*P-P: peak-to-peak airway pressure, representing the maximal change between inspiratory and expiratory airway pressure. The higher the resistive load of the device, the higher the *P-P*

The test lung simulated moderate and high respiratory demands: different muscle pressures were simulated to target tidal volumes (Vt) of 300 and 500 mL. *P-P* is defined as the maximum change between inspiratory and expiratory airway pressure throughout the simulated respiratory cycle. It represents the ability of the device to maintain the positive airway pressure at the level set regardless of the phase of the respiratory cycle, the ideal value being equal to zero. The Bag-CPAP was tested in different oxygen flow conditions, with the Venturi connector at 5 L/min and 10 L/min and with the conventional oxygen connector at 15 L/min and 20 L/min. All differences between devices were statistically significant (one-way ANOVA with Bonferroni correction, *p* < 0.005)

Relative changes in WOB of the simulated patient required to maintain the tidal volume with each device compared with those observed with PB980 ventilator (ΔWOB expressed as a percentage of WOB observed with PB 980 ventilator) are reported in Table 4. AirSense CPAP required lower WOB than PB980 ventilator to achieve the target tidal volume (either 300 or 500 mL), whereas the other systems exhibited higher WOB than PB980 ventilator. Overall, WOB was lower with virtual valve CPAPs (Boussignac, O-two) compared with Venturi type CPAP (Ventumask, Bag-CPAP). The Bag-CPAP exhibited the highest value of ∆WOB with 5 L/min oxygen flow rate (Venturi) for both demands. Interestingly, increasing its oxygen flow rate to 10 L/min (Venturi) significantly decreased ∆WOB resulting in lower resistive load than those of open valve systems. Additional file 1: Table S2 illustrates the oxygen flow needed to obtain a comparable WOB with Boussignac CPAP and Bag-CPAP. All results displayed in Table 4 are illustrated in Additional file 1: Fig. S3 using dynamic Pmus/volume loops.

**Table 4:** Calculation of the ∆WOB patient (%) according to two simulated inspiratory demands

	Moderate inspiratory demand with Vt target of 300 ml		High inspiratory demand with Vt target of 500 ml	
	∆WOB patient (%)		∆WOB patient (%)	
PB 980	0		0	
AirSense CPAP	− 2		− 11	
Boussignac CPAP	+ 35		+ 26	
O-Two CPAP	+ 50		+ 38	
Ventumask CPAP	+ 68		+ 96	
Bag-CPAP with Venturi^a^	+ 137	+ 18	+ 106	+ 18
Bag-CPAP with conventional connector^b^	+ 92	+ 37	+ 71	+ 66

^a^Oxygen flow set at 5 L/min and 10 L/min

^b^Oxygen flow set at 15 L/min and 20 L/min

**Fig. 4:**
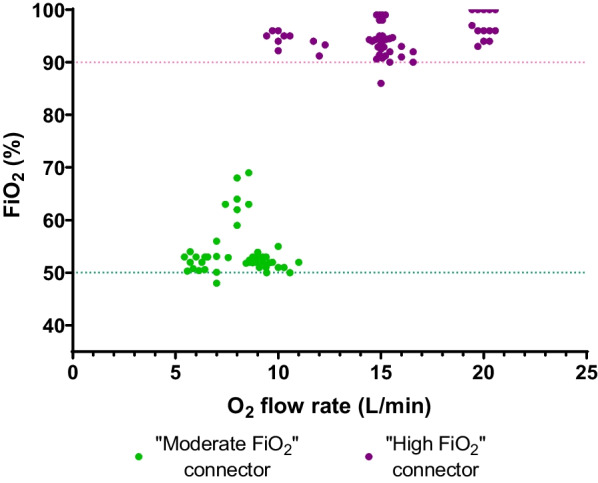
Actual fraction of inspired oxygen (FiO_2_) delivered by Bag-CPAP in patients with acute hypoxemic respiratory failure. FiO_2_ measured into the mask in patients on Bag-CPAP treatment according to the oxygen flow rate. Each circle represents one measurement. Green circles denote the use of the “moderate FiO_2_” Venturi connector whereas the violet circles denote the use of the “high FiO_2_” connector

*∆WOB patient* represents the relative variation of the simulated patient’s work of breathing needed to maintain the tidal volume obtained with the ICU ventilator (PB 980). See Fig. 4 as well. Bag-CPAP was tested in different oxygen flow conditions, with the Venturi connector at 5 L/min and 10 L/min and with the conventional oxygen connector at 15 L/min and 20 L/min

### Clinical study

Ten men and ten women with a median age of 70 (61–87) years treated with Bag-CPAP for AHRF were included in the clinical study. The etiologies of AHRF included SARS-CoV-2 related pneumonia (*n* = 14), acute cardiogenic pulmonary edema (*n* = 4) and acute exacerbation of interstitial pneumonia (*n* = 2).

#### Technical endpoints

A hundred FiO_2_ recordings were obtained, of which 45 with the “moderate FiO_2_” connector and 55 with the “high FiO_2_” one. The “moderate FiO_2_” connector coupled with a median oxygen flow rate of 8 (7–9) L/min generated a median FiO_2_ delivered into the mask of 52 (51–53) % (Fig. 4). The “high FiO_2_” connector used with a median oxygen flow rate of 15 L/min (15–16) generated a median FiO_2_ of 94% (93–96) into the mask (Fig. 4). The median PEEP recorded into the mask was 6.5 (5.3–7.0) cm H_2_O and the median peak-to-peak airway pressure was 4 (3–5) cm H_2_O.

#### Physiological endpoints and clinical course

**Fig. 5:**
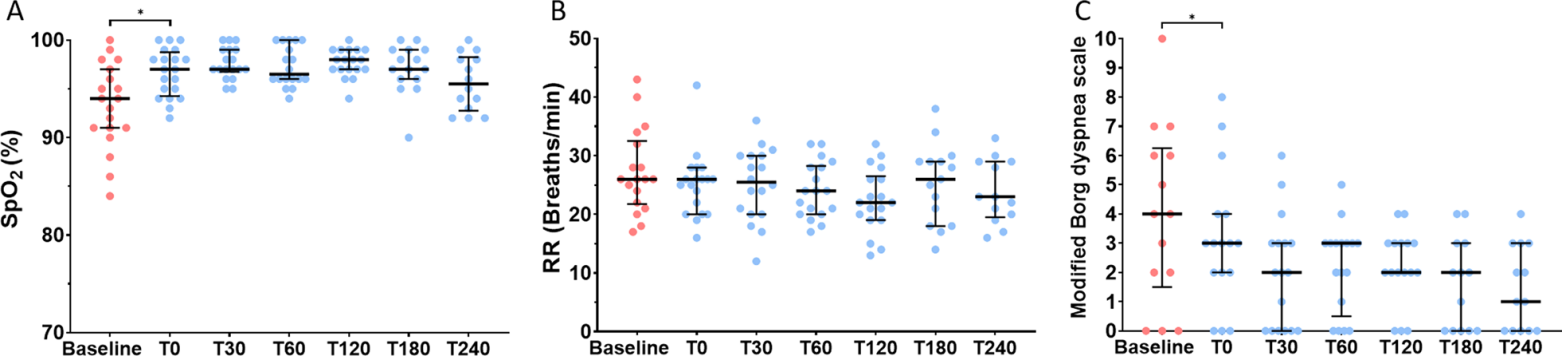
Evolution of pulse oximetry (SpO_2_), respiratory rate (RR), and modified Borg dyspnea scale after initiation of Bag-CPAP in patients with acute hypoxemic respiratory failure. Readings of SpO_2_ (panel **A**), respiratory rate (panel **B**), and modified Borg dyspnea scale (panel **C**) were collected at baseline under conventional oxygen therapy before Bag-CPAP initiation (red circles), within 10 min after Bag-CPAP initiation (T0; blue circles), and after 30, 60, 120, 180, and 240 min of Bag-CPAP treatment (T30, T60, T120, T180 and T240, respectively; blue circles). * denotes *p value* < 0.05

Bag-CPAP initiation led to a significant increase in SpO_2_ [from 93% (91–97) to 97% (95–99), *p* < 0.001] and a significant decrease in the dyspnea score [from 4 (2–7) to 3 (2–4), *p* = 0.017], without affecting the respiratory rate (Fig. 5). None of the physiological effects observed upon Bag-CPAP initiation changed over the first 240 min of follow up (Fig. 5). In general, Bag-CPAP was well tolerated in all but one patient who previously received bilevel noninvasive ventilation in the emergency department for cardiogenic pulmonary edema prior to Bag-CPAP initiation. That patient complained of “air hunger” presumably due to loss of pressure support, thus was put on bilevel noninvasive ventilation again. Eight patients (SARS-CoV-2 related pneumonia, *n* = 7; acute exacerbation of interstitial pneumonia, *n* = 1) were eventually intubated after a median of 2 [1–3] days, and seven patients (SARS-CoV-2 related pneumonia, *n* = 6; acute exacerbation of interstitial pneumonia, *n* = 1) eventually died in ICU.

## Discussion

We herein report a comprehensive bench-to-bedside study of a frugal CPAP specifically adapted to oxygen delivery constraints. On the bench, Bag-CPAP had the highest oxygen delivery efficiency, whereas the homecare ventilator CPAP had the lowest resistive properties. The clinical study confirmed the ability of Bag-CPAP to deliver FiO_2_ of 50–60% or > 90% with oxygen flow rates of 5–15 L/min, respectively, according to patient’s needs. Despite the high resistive properties measured on the bench with low oxygen supply, the clinical tolerance was considered acceptable based on Borg scales showing an overall decrease in dyspnea over the course of the treatment.

### Oxygen delivery constraints

For the first time since the polio epidemics in the 50 s, many healthcare facilities have been overwhelmed by the significant pressure that impacted their capacity to properly manage surges of COVID-19 patients [14]. Moreover, the unprecedented number of hypoxemic patients requiring high FiO_2_ has threatened the oxygen supply and occasionally created shortages [15]. In specific geographic areas such as India, the use of oxygen concentrators delivering a limited oxygen flow rate of 5–10 L/min was necessary to compensate for the lack of liquid oxygen [3]. This unanticipated scenario has changed the paradigm of oxygen management demonstrating for the first time the necessity to think oxygen supply logistic differently and to consider medical devices dedicated to noninvasive oxygen administration in high income countries as well. The wide use of high flow oxygen therapy (HFOT), although efficient to alleviate respiratory distress and to manage gas exchange, might have jeopardized oxygen supplies by generating unexpected considerable oxygen consumption [16]. In the meantime, noninvasive CPAP, partially abandoned for years after the negative results reported by Delclaux et al. in hypoxemic respiratory failure, has recently proved to be as efficient as HFOT therapy to treat COVID-19-related severe respiratory failure [6, 17]. Even if the literature evidence is still incomplete, CPAP could be considered as an alternative to HFOT to noninvasively treat hypoxemic respiratory failures. Even before the pandemic, limited oxygen delivery and shortages had already been part of the landscape in low-middle income countries, which host the majority of critically-ill patients requiring respiratory support on earth, but where this resource is structurally scarce, leading to a considerable loss of opportunity for patients [4]. The frugal innovation approach allows to incorporate these constraints as a *prima materia* when designing new devices for noninvasive respiratory support.

### Working principles of Bag-CPAP

The main purpose of the present study was to test a new original CPAP device frugally engineered to provide moderate (50–60%) or high (> 90%) FiO_2_ irrespective of the patient’s inspiratory demand, while minimizing oxygen consumption. It combines two main working principles: a buffer reservoir and a Venturi system.

The 30L reservoir of Bag-CPAP is working as a buffer to help maintain constant FiO_2_ even with high respiratory demand. Additionally, the reservoir accumulates oxygen continuously, limiting the loss of oxygen usually insufflated during expiration with most conventional systems (e.g., open valve CPAP). Based on a similar approach, a spring expiratory positive pressure valve connected to an elastic reservoir was described several years ago, but the purpose of that old reservoir-based system was mainly to improve positive pressure during inspiration rather than to optimize FiO_2_/oxygen consumption balance [18]. Moreover, that system was not adapted to noninvasive ventilation unlike the Bag-CPAP reservoir which remains inflated for a couple of minutes even in case of massive leaks or mask disconnection.

As observed in our results, the specifically designed Venturi system significantly reduced oxygen consumption while allowing to reach and maintain FiO_2_ above 50%. The oxygen flow drives into a larger conduit, generating a decrease of pressure that absorbs external air and creates a mixture of air-oxygen at a higher flow [19]. The Venturi system of Bag-CPAP was frugally designed to specifically meet the clinical needs observed in real life, i.e., to optimize oxygen consumption [4]. Interestingly, FiO_2_ measured under experimental conditions reached the two predefined ranges (40–60% and 90–100%) with a minimal oxygen flow of 5 and 15 L/min, respectively, which makes Bag-CPAP the device with the highest oxygen saving properties among the tested systems. These valuable performances should be balanced by a higher resistive load when the oxygen flow is limited. However, this resistive load could be significantly reduced upon increasing oxygen flow, whenever possible. Moreover, at moderate FiO_2_ obtained with Bag-CPAP and the Venturi connector, the WOB measured with 10 L/min of oxygen flow rate is similar to the WOB measured with Boussignac valve at 30 L/min (see Additional file 1: Table S2). Overall, under constrained conditions, Bag-CPAP is the most oxygen delivery-efficient device which guarantees reaching and maintaining the targeted FiO_2_ at the cost of an increased resistive workload.

### Comparators

The concept of open valve system was initially developed by Dr Georges Boussignac to provide CPAP through a very simple single-use disposable device powered by a low pressure oxygen source. Bench studies have demonstrated the low additional work induced by open valves and their ability to maintain CPAP level generated even during inspiration [5]. Of note, open valves outperformed Bag-CPAP in terms of pressure control during inspiration. However, open valve systems were not able to guarantee stable FiO_2_ levels since ambient air contaminated oxygen actually delivered to the patient when the inspiratory demand significantly increased. Another limitation is that both PEEP and FiO_2_ generated by the open valve are directly related to oxygen flow supply.

Oxygen supplied via a Venturi system directly connected to a mask might be a good alternative as it is the case with Ventumask. Nevertheless, this system needs a high oxygen flow rate to reach high FiO_2_ and it does not spare oxygen wasted during expiration, unlike Bag-CPAP. As expected, the homecare turbine ventilator accurately regulated the positive pressure, exhibited a low resistive load, with relatively low oxygen consumption, and perfectly reached the FiO_2_ targets even if the low pressure oxygen source coupled with the turbine theoretically limits the ability to maintain FiO_2_ when inspiratory demand rises. Overall, as compared with other systems, Bag-CPAP had the highest oxygen saving properties, allowing to treat almost three times more patients, i.e., with comparable FiO_2_ delivered while using similar oxygen input.

### Clinical implications

Based on a composite outcome criteria, a recent large randomized controlled trial demonstrated that CPAP might be as efficient as HFOT in managing patients admitted to the emergency department with COVID-19-related AHRF [6]. Our study showed the heterogenic bench performances of the various CPAP systems which may also differ in terms of ease of use and training.

The high oxygen saving properties of Bag-CPAP were confirmed in the clinical study. Bag-CPAP maintained FiO_2_ above 50% with oxygen flow rates ranging from 5 to 10 L/min, hence the possibility to use it with oxygen concentrators as an alternative to pressurized oxygen supply. The PEEP valve used in the present clinical evaluation was limited to 7.5 cm H_2_O. Increasing PEEP would require the use of a different single-use PEEP valve offering the possibility to manually adjust PEEP from 5 to 15 cm H_2_O. Despite the high resistive load observed in vitro, the clinical tolerance of Bag-CPAP assessed by Borg scales and SpO_2_ evolution was acceptable. A single patient, previously treated with bilevel noninvasive positive pressure ventilation, asked to interrupt Bag-CPAP. In this small series, the intubation rate was 40%, which falls within the range recently reported in patients with COVID-19-related acute hypoxemic respiratory failure treated with noninvasive respiratory supports [6]. Clinical studies are warranted to compare head to head Bag-CPAP and other devices in terms of efficacy and tolerance.

### Strengths and limitations of the study

The main strength of our study lies in its completeness, from bench to bedside. Our study has several limitations. First, the devices selected for the bench study did not include all available CPAP systems. The tested devices were selected to compare different technologies and working principles facing specific oxygen delivery constraints, as experienced during the COVID-19 crisis or in low-middle income countries. Second, the small number of patients enrolled in the clinical study as well as the non-comparative study design did not allow to draw conclusions regarding clinical superiority or equivalence of Bag-CPAP compared to other treatments. A larger randomized controlled study is necessary to confirm the ability of the device to treat severe AHRF patients with whom the Bag-CPAP device presents limitations. The size and weight of the device attached to the mask could limit the tolerance of the technique compared with lighter systems such as open-valve CPAP (see Additional file 1: Table S3). Third, to measure FiO_2_ actually delivered to patients, an external FiO_2_ sensor is required, thus limiting the possibility of closely monitoring PaO_2_/FiO_2_ ratio. Of note, FiO_2_ was not systematically measured before initiation of Bag-CPAP. In addition, the level of PEEP was slightly lower than expected due to the working principle of the spring PEEP valve or the way we measured PEEP in the mask under dynamic clinical conditions. Eventually, as Bag-CPAP was specifically designed to address constrained situations or environments, its assessment in these specific situations is warranted.

## Conclusion

Bag-CPAP device exhibited the highest oxygen saving properties compared with other devices tested in the bench experiment, albeit with an increased resistive load. Although non-comparative, clinical observations suggested an acceptable clinical tolerance while limiting oxygen consumption as compared with commercially available CPAP devices. Additional physiological studies are necessary to confirm the clinical benefit expected with this Bag-CPAP in a constrained environment.

## Supplementary Information

**Additional file 1.** Additional methods and results.

## Data Availability

The datasets used and/or analyzed in the current study are available from the corresponding author on reasonable request.
