# Supplementary material for: A new reservoir-based CPAP with low oxygen consumption: the Bag-CPAP
Source: Crit Care. 2023 Jul 4;27:262. doi: 10.1186/s13054-023-04542-2 (PMC10318806; doi:10.1186/s13054-023-04542-2)
Supplement: Supplementary file 1 — Additional file 1. Additional methods and results. [file 13054_2023_4542_MOESM1_ESM.docx]

# Additional file

# Additional methods

## Volume averaged FiO_2_ calculation

The volume averaged FiO_2_ estimates if there is a match between the peak inspiratory flow and the actual delivered FiO_2_. Volume averaged FiO_2_ represents the effective FiO_2_ delivered to the lungs. The oxygen flow passing through the trachea over time is calculated by multiplying the patient’s inspiratory flow (Q_patient_(t)) by the FiO_2_ measured at the same time point [Equation 1].

*Equation 1:* $oxygen flow in the trachea=Q_{patient}\left( t \right).F{iO}_{2}(t)$

The start and the end of the patient’s inspiratory time were identified when the patient’s flow rate crossed 0 L/s. The oxygen flow needs to be integrated over time to determine the actual volume of oxygen in inspiration. [Equation 2].

*Equation 2:* $inspired volume of oxygen=\int_{Ti0}^{Ti} Q_{patient}\left( t \right).F{iO}_{2}\left( t \right)dt$

Volume-averaged FiO_2_ is finally obtained by calculating the ratio of inspired volume of oxygen divided by tidal volume (3).

*Equation 3:*

$Volume averaged FiO_{2}=\frac{\int_{Ti0}^{Ti} Q_{patient}\left( t \right).FiO_{2}\left( t \right)dt}{Vt} with Vt=\int_{TiO}^{Ti} Q_{patient}\left( t \right)dt$

## Implementation of a tutorial video to train medical teams

Before Bag-CPAP implementation in healthcare services, the staff received a standardized training through a 6-min video tutorial (https://www.youtube.com/watch?v=GVnwCY7An4E) completed by bedside teaching from investigators if needed. This tutorial was available via a QR code placed in the staff room and on the user manual distributed with Bag-CPAP. This user manual presented how to install Bag-CPAP on a patient in three easy steps.

The implementation of Bag-CPAP in clinical practice within the hospitals participating in the present study was straightforward without obstacles. The absence of misuse or incorrect installation reports in both centers indirectly suggested the simplicity of implementation of Bag-CPAP at the bedside by untrained caregivers, a relevant aspect for a frugal medical device.

## Supplementary Figure 1. Schematic description of the volume averaged FiO_2_ calculation criteria in two illustrative situations

**
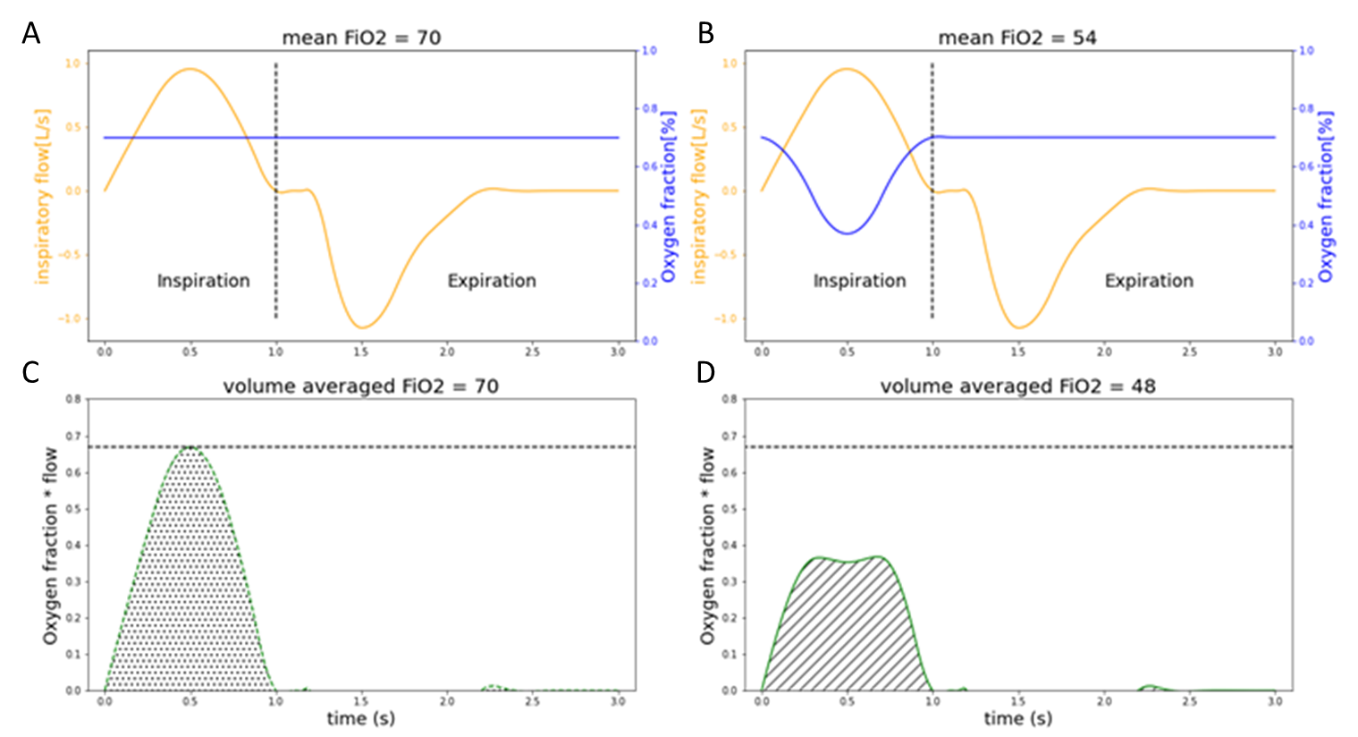
**

Panels A and B represent oxygen flow and inspired fraction (FiO_2_) measured in a single breath. On these panels, the yellow curves represent the patient’s spontaneous respiratory flow in one breath whereas the blue ones illustrates the FiO_2_ measured in the mask of the patient during the same breath time. Panels C and D present a visualization of the respiratory flow multiplied by FiO_2_ according to time. The area under the curve is proportional to the volume averaged FiO_2_. In B and D panels, the FiO_2_ rate drops when the patient inspiratory flow increases (e.g. open-valve CPAP devices behavior). Consequently, the oxygen molecule rate received by the patient over the inspiratory time also decreases. This method takes into account the patient’s flow/FiO_2_ relative behavior in the volume averaged FiO_2_.

# Additional Results

## Supplementary Figure 2. FiO_2_ recorded on the bench with different tested devices at moderate and high FiO_2_ targets

**
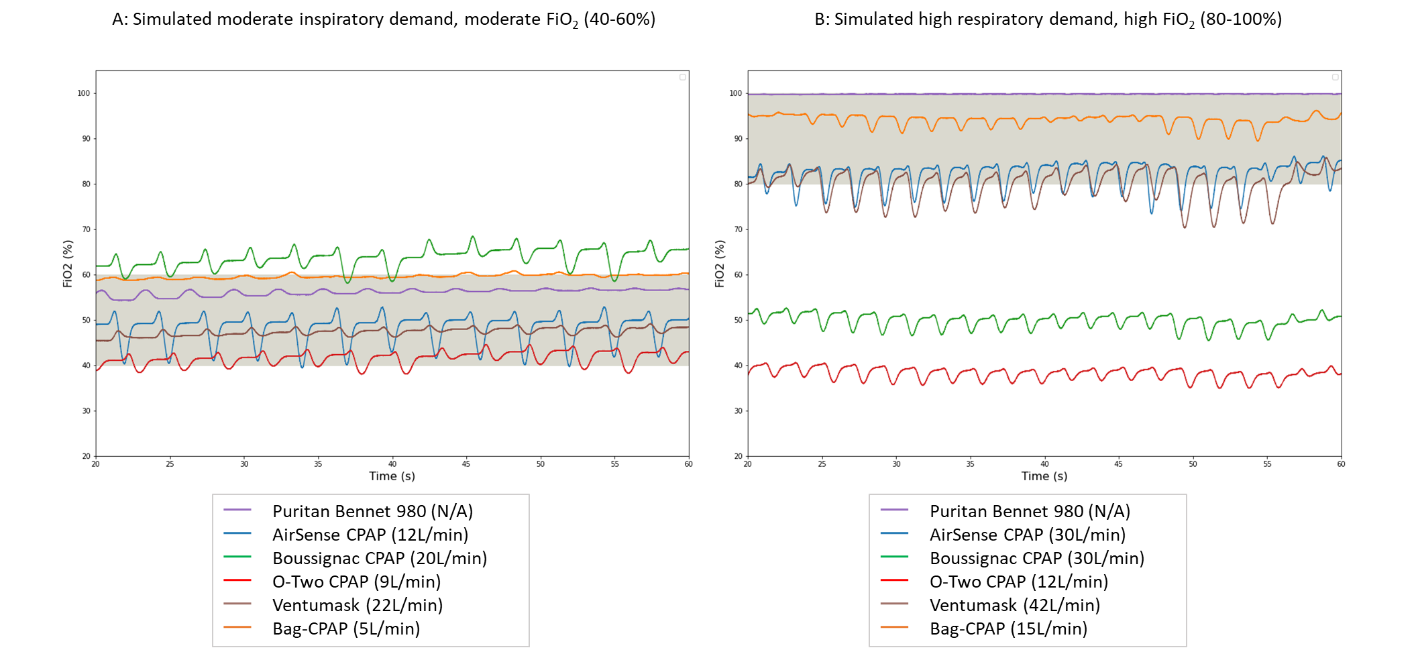
**

FiO_2_ actually delivered by each device was assessed at two FiO_2_ target ranges corresponding to two clinical scenario: 40 to 60% while the test lung simulated a moderate respiratory demand (panel A) and 80 to 100% while the test lung simulated a high respiratory demand (panel B). Oxygen flow rates were adjusted on each device according to manufacturer’s recommendations to reach those two target ranges whilst trying to deliver a PEEP level between 5 and 10 cm H_2_O.

The areas shaded in grey represent the two FiO_2_ target ranges.

## Supplementary Figure 3. Pressure volume curves obtained on the bench to compare additional resistive work induced by the continuous positive airway pressure devices.

**
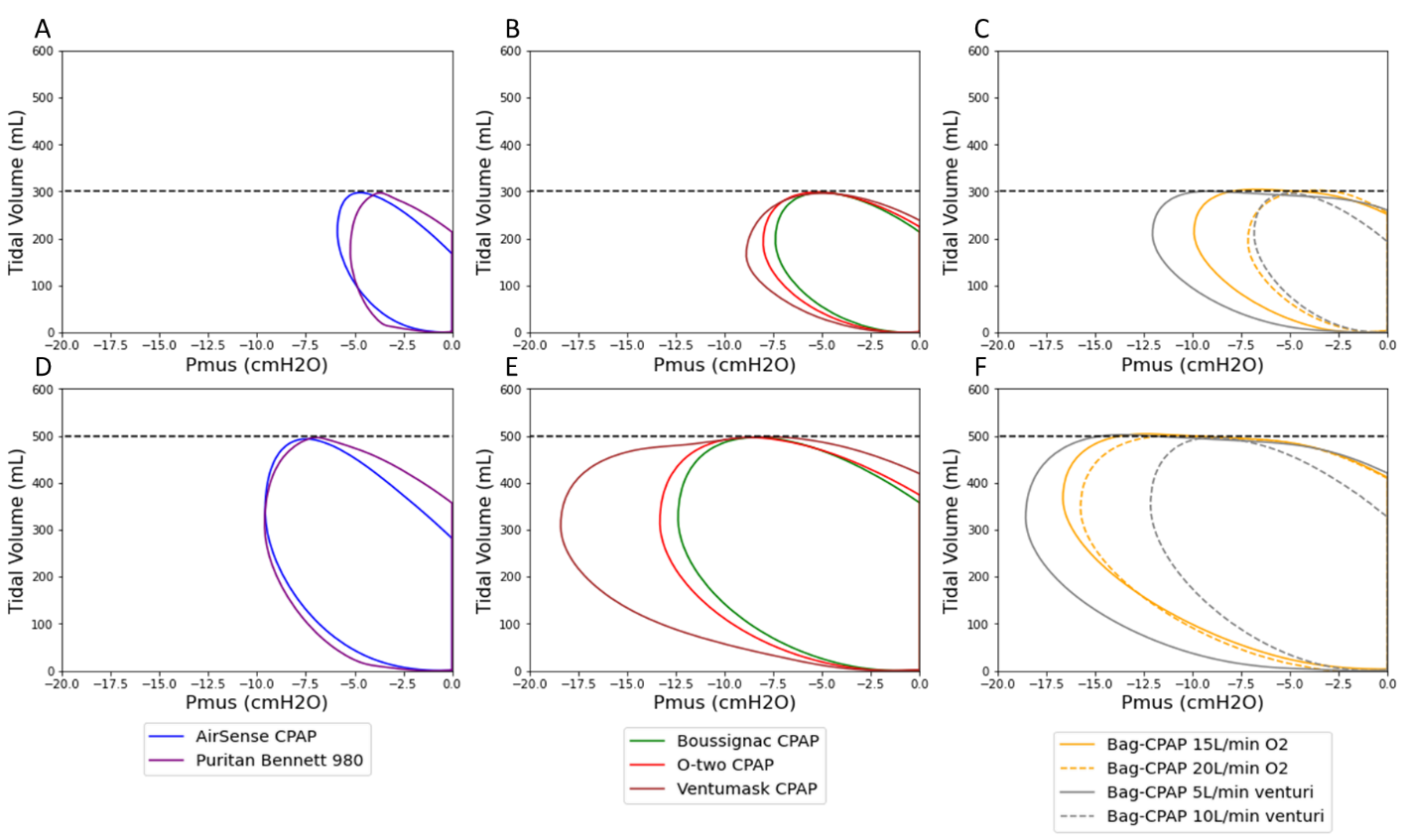
**

The figure represents dynamic simulated muscle pressure (Pmus)/volume loops recorded with each device. The pressure/volume loops for all devices were obtained by adjusting the simulated patient’s effort (Pmus) to reach a given tidal volume. Panels A, B, and C represent the Pmus/volume loops with moderate respiratory demand (Rrs = 6 cm H_2_O/L/s, Crs = 60 mL/cm H_2_O, RR = 20 cpm) targeting a tidal volume of 300 mL. Panels D, E, and F represent the Pmus/volume loops with high respiratory demand (Rrs = 6 cm H_2_O/L/s, Crs = 60 mL/cm H_2_O, RR = 20 cpm) targeting a tidal volume of 500 mL. Work of breathing (WOB) was defined as the trapezoidal numerical integration of the Pmus/volume curve, which corresponds to the area under the curve. The bigger the area, the higher the WOB imposed by the device. The relative difference in the areas represents the change in WOB (∆WOB patient) imposed by the device to keep the tidal volume constant against a reference. PB980 ventilator was defined as the reference and ∆WOB was computed for each device (see Table 2).

# Supplementary Tables

## Supplementary Table 1. General characteristics of the tested devices

|  | **Puritan Bennett 980** | **AirSense**  **10Autoset** | **Boussignac CPAP** | **O-Two CPAP** | **StarMed Ventumask** | **Bag-CPAP** |
| --- | --- | --- | --- | --- | --- | --- |
| **Manufacturer** | Covidien | Resmed | Vygon | O-Two | Intersurgical | Air Liquide Medical Systems |
| **Gas mixture** | Pneumatic | Turbine | Open valve | Open valve | Venturi | Venturi |
| **PEEP range** (cmH_2_O) | 0-45 | 4-20 | 5-10 | 5-25 | 5-12.5 | 7.5-10 |
| **FiO_2_ range** (%) | 21-100 | N/A | >50% | 54-77 | 40-100 | 50 or >90 |
| **Oxygen flow rate** (L/min) | 0-200 | N/A | 20-30 | 8-25 | 12-42 | 5-20 |
| **NIV interface** | Respireo Hospital^1^ | AirFit F10^2^ | Respireo Hospital | Respireo Hospital | Ventumask^3^ | Respireo Hospital |

Abbreviations: PEEP: positive end expiratory pressure, FiO_2_: inspired fraction of oxygen, NIV: noninvasive ventilation

^1^ Air Liquide Medical Systems, Brescia, Italy; ^2^ Resmed, Saint Priest, France; ^3^ Intersurgical, Wokingham, United Kingdom

## Supplementary Table 2. Bench comparison of Bag-CPAP with Boussignac CPAP to obtain comparable WOB.

| **Device** | **Oxygen flow (L/min)** | **∆WOB (%)** |
| --- | --- | --- |
| Boussignac | 30 | 0 |
| Bag-CPAP with Venturi connector | 10 | +8 |
| Bag-CPAP with conventional connector | 30 | +5 |

 This table presents the performances of Boussignac CPAP and Bag-CPAP with two oxygen flow rates, and shows comparable WOB (difference less than 10%). The table compares Boussignac CPAP at 30 L/min with Bag-CPAP in two conditions (10 L/min oxygen, with Venturi connector and 30 L/min oxygen with conventional oxygen connector). Results show that to obtain comparable WOB, Bag-CPAP requires three times less oxygen flow to reach the moderate FiO_2_ target (50-60%) and the same flow to reach the high FiO_2_ target (> 90%). Therefore, Bag CPAP is versatile in terms of uses: i) in case of oxygen shortage, Bag-CPAP can provide high FiO_2_ while preserving oxygen, at the expense of increased resistive work; ii) in less constrained conditions, increasing the oxygen flow rate of Bag-CPAP can help reduce its resistive work while maintaining high FiO_2_ regardless of the patient’s inspiratory effort.

## Supplementary Table 3. Weights of the tested noninvasive devices

| **Device (+filter)** | **Weight (g)** |
| --- | --- |
| Boussignac CPAP | 261 |
| O-two CPAP | 381 |
| Ventumask CPAP* | 1191 |
| Bag-CPAP | 556 |

*The weight of Ventumask includes the NIV interface.
